# Computational Studies on the Substrate Interactions of Influenza A Virus PB2 Subunit

**DOI:** 10.1371/journal.pone.0044079

**Published:** 2012-09-05

**Authors:** Ya-Jun Wang, Jing-Fang Wang, Jie Ping, Yao Yu, Ying Wang, Peng Lian, Xuan Li, Yi-Xue Li, Pei Hao

**Affiliations:** 1 Key Laboratory of Systems Biomedicine (Ministry of Education), Shanghai Jiao Tong University, Shanghai, China; 2 Shanghai Center for Bioinformation and Technology, Shanghai, China; 3 Bioinformatics Center, Key Laboratory of Systems Biology, Chinese Academy of Sciences, Shanghai, China; 4 Institute of Plant Physiology and Ecology, Chinese Academy of Sciences, Shanghai, China; 5 Institute of Pasteur, Shanghai Institutes for Biological Sciences, Chinese Academy of Sciences, Shanghai, China; University of Lethbridge, Canada

## Abstract

Influenza virus, which spreads around the world in seasonal epidemics and leads to large numbers of deaths every year, has several ribonucleoproteins in the central core of the viral particle. These viral ribonucleoproteins can specifically bind the conserved 3′ and 5′ caps of the viral RNAs with responsibility for replication and transcription of the viral RNA in the nucleus of infected cells. A fundamental question of most importance is that how the cap-binding proteins in the influenza virus discriminates between capped RNAs and non-capped ones. To get an answer, we performed molecular dynamics simulations and free energy calculations on the influenza A virus PB2 subunit, an important component of the RNP complexes, with a cap analog m7GTP. Our calculations showed that some key residues in the active site, such as Arg355, His357, Glu361 as well as Gln406, could offer significant hydrogen bonding and hydrophobic interactions with the guanine ring of the cap analog m7GTP to form an aromatic sandwich mechanism for the cap recognition and positioning in the active site. Subsequently, we applied this idea to a virtual screening procedure and identified 5 potential candidates that might be inhibitors against the PB2 subunit. Interestingly, 2 candidates Cpd1 and Cpd2 have been already reported to have inhibitory activities to the influenza virus cap-binding proteins. Further calculation also showed that they had comparatively higher binding affinities to the PB2 subunit than that of m7GTP. We believed that our findings could give an atomic insight into the deeper understanding of the cap recognition and binding mechanism, providing useful information for searching or designing novel drugs against influenza viruses.

## Introduction

Influenza, commonly referred to the flu, is an acute viral-infection disease caused by a number of RNA viruses of the family Orthomyxoiridae (also known as influenza viruses) [1]. Typically, influenza viruses are transmitted through the air by coughs or sneezes, creating aerosols containing the viruses, or through direct contact with bird droppings or nasal secretions, or through contact with contaminated surfaces [2], [3]. Nowadays, influenza virus spreads around the world in seasonal epidemics, leading to 25,000–500,000 deaths every year, which will be up to millions in the pandemic years [4], [5]. Although having a number of subtypes, influenza viruses share a similar overall structure: the virus particle is roughly spherical with a diameter of about 80–120 nm [6]. The viral envelope contains a proton channel and two glycoproteins, wrapped around the central core, which contains the viral RNA genome and other viral proteins [7], [8].

In the past few years, some powerful antiviral drugs have been developed to treat and prevent influenza infection targeted on the proteins in the viral envelope [9], [10], [11], [12]. These antiviral drugs can be clustered into two major types: neuraminidase inhibitors (i.e., oseltamivir and zanamivir) and proton channel inhibitors (i.e., amantadine and rimantadine). Currently, neuraminidase inhibitors are preferred for influenza virus infections since they are less toxic and more effective [13]. However, increased resistance has been detected in patients with this kind of antiviral drugs [14], [15]. Since then, a series of good attempts have been made by experimental and theoretical approaches to study the structural mechanism of drug inhibition and resistance for these antiviral drugs, with an aim of searching for an effective approach to prevent the known drug resistance [16]–[21]. However, to avoid the known resistance, an alter strategy is to develop novel antiviral drugs targeting on other proteins (or RNA) in the central core of influenza viruses, i.e., the polymerase complex of influenza viruses that is found to be essential for viral replication.

For influenza A viruses, the viral genome in the central core of the viral particle contains 8 single-stranded RNA segments of negative polarity with partially complementary ends, encoding totally 11 important viral proteins. Each single-stranded RNA segment can form several ribonucleoprotein (RNP) complexes via the association with multiple monomers of the nucleoprotein (NP) and one single copy of the viral RNA-dependent RNA polymerase composed of three subunits: one polymerase acidic protein PA, and two polymerase basic proteins PB1 and PB2 [22], [23]. The RNP complexes can bind the conserved 3′ and 5′ caps of each viral RNA segment, and are responsible for replication and transcription of the viral RNA in the nucleus of infected cells. Host-cell pre-mRNA is bound to the PB2 subunit by its 5′ caps, which is also considered as the initial step of viral mRNA transcription [24], [25].

In 2008, Guilligay and his co-workers released an atomic-resolution structure of influenza A virus PB2 cap binding domain (residues 318–483) with bound m7GTP and provided functional analysis to show that the cap-binding site is essential for cap-dependent transcription by viral RNPs in vitro and in vivo [26]. They also suggested that PB2 cap binding domain is structurally distinct from other cap-binding proteins, and will be a good target for developing novel antiviral drugs. However, deeper understanding of the structural flexibility and its interactions with 5′ cap RNAs is still needed. In comparison with the crystal and EM studies of the influenza virus PB2 subunit, computational approaches, especially molecular dynamics (MD) simulations and free energy calculations, have advantages to analyze the conformational fluctuations of the PB2 subunit and its interactions with RNAs [27], [28], [29]. In the current study, we employed MD simulations and free energy calculations to give an atomic description to the interactions of the influenza A virus PB2 subunit with cap analog m7GTP. Based on these structural analyses, we would provide a novel strategy for antiviral drug screening, which was further used to search for the potential leading compound in the related databases.

## Materials and Methods

### Initial structure and MD simulations

By now, only 1 crystal structure (2vqz.pdb) of the influenza A virus PB2 subunit is available in the Protein Data Bank [26], which was thus selected as the initial structure for the further calculations. Except for the polar hydrogen and heavy atoms of the protein and the ligand, all the other atoms including non-polar hydrogen in the crystal structure and the crystal water molecules were deleted. The pKa values for each residue in the influenza A virus PB2 subunit were computed using Delphi [30] as a Poisson-Boltzmann solver with a dielectric constant of 4. Hydrogen atoms were then added to the protein structure with t-Leap procedure in AMBER 11 package [31] based on the computational pKa values. Subsequently the simulation systems were solvated in a simulation box with explicit TIP3P water models. To neutralize the solvated systems, chloride ions were added to random place equal number of water molecules in the simulation box. All the atoms of the influenza A virus PB2 subunit and its ligands were parameterized by Amber force field parameters [32].

After solvation, the simulation systems were subjected to steepest descent energy minimization for about 5000 steps followed by conjugate gradient for the next 5000 steps, and then equilibrated with the atoms in the protein and ligand fixed by a series short MD simulation (∼1 ns) to reduce the van der Waals conflicts in the simulation systems. Finally, 10-ns MD simulations were performed under the normal temperature (310 K) by AMBER 11 package [31] with periodic boundary conditions and NPT ensemble. The SHAKE algorithm with a tolerance of 10^−6^ was used to constrain all bonds in the simulation systems involving hydrogen atoms, and atomic velocities for start-up runs were obtained according to the Maxwell distribution at 310 K. 10 independent simulation trajectories with different starting vectors were for each simulation system were generated. The isothermal compressibility was set to be 4.5×10^−5^ per bar for solvent simulations. The electrostatic interactions were treated by PME algorithm with interpolation order of 4 and a grid spacing of 0.12 nm. The van der Waals interactions in our study were computed by using a cut-off value of 12 Å. All the MD simulations were run with a time step of 2 fs, and coordinates for the simulation systems were saved every 1 ps.

### Free energy calculation

The molecular mechanics Poisson-Boltzmann surface area (MM-PB/SA) and Generalized Born surface area (MM-GB/SA) approaches [33] implemented in AMBER 11 package was applied to calculate the binding affinities for m7GTP and other ligands. Such approaches have been widely used to study protein folding [34], [35], protein-DNA (or RNA) interactions [36], [37], protein-ligand interaction [38], [39], [40], as well as structure-based drug design [41], [42], [43]. The principles of both MM-PB/SA and MM-GB/SA approaches can be summarized as following:
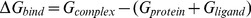
(1)

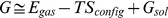
(2)

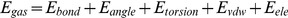
(3)

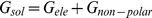
(4)In equation 1, the binding free energy change (Δ*G*) is computed as the difference between the free energies of the complex (*G_complex_*), the protein (*G_protein_*) as well as the ligand (*G_ligand_*). These free energies are calculated through equation 2 by summing up its internal energy in the gas phase (*E_gas_*), the solvation free energy (*G_sol_*), and a vibrational entropy term (*T*Δ*S*). The simulation systems involved in our study contained ∼150,000 atoms, while the variance of the atom number for different ligands was less than 100, indicating that the entropy differences for the same protein with different ligands was induced by less than 0.07% of the total atoms in the simulation system. Thus, we thought that the same protein with different ligands employed almost the same entropy, and the entropy contributions in the free energy calculations are neglected. *E_gas_* is a standard force field energy calculated from equation 3 by the strain energies from covalent bonds (*E_bond_* and *E_angle_*) and torsion angles (*E_torsion_*), non-covalent van der Waals (*E_vdw_*), as well as electrostatic energies (*E_ele_*). As described in equation 4, the solvation free energy (*G_sol_*) is calculated by both an electrostatic term (*G_ele_*) and a non-polar component (*G_non-polar_*). The former can be obtained from either the Possion-Boltzmann (PB) approach or Generalized Born (GB) approach, while the latter is considered to be proportional to the molecular solvent accessible surface area. In our study, totally 200 snapshots retrieved from the last 1-ns segment on the MD trajectories with an interval of 5 ps were used for calculating the binding free energies.

### Virtual screening procedure and multi-target selectivity

The cap analog m7GTP was selected as a structural template to search our in-house database which contained all the bioactive drug-like small molecules in ChEMBL and CNPD (Chinese Natural Product Database, Neotrident Technology Ltd., China). MACCS keys [43] were employed to find the molecules that had highly similarity with the template m7GTP, and those having high similarity with the template m7GTP were further assessed by Lipinski's Rule Five [44]. The candidates in accordance with Lipinski's Rule Five were then subject to a flexible docking procedure using AutoDock 4.0 package, and the ones employed similar or higher docking scores were picked up for the molecular dynamics simulations and free energy calculations. During the docking procedure, a grid box with dimensions of 128×128×128 cubic angstroms was used, and rotatable bonds were allowed for the candidates to rotate during docking operations. For each docking operation, 100 runs with 10 million energy evaluations were carried out using Lamarkian genetic algorithm.

The candidates found in the aforementioned procedures were then subjected to the multi-target selectivity studies. Totally 841 known drug targets were used in this multi-target selectivity procedure, which were collected from the Potential Drug Target Database (PDTD) [45]. The candidates were docked into all the targets using AutoDock 4.0 with Lamarkian genetic algorithm. The final results for each candidate were listed according to their docking scores.

## Results and Discussion

### Trajectory analysis and free energy calculation

**Figure 1:**
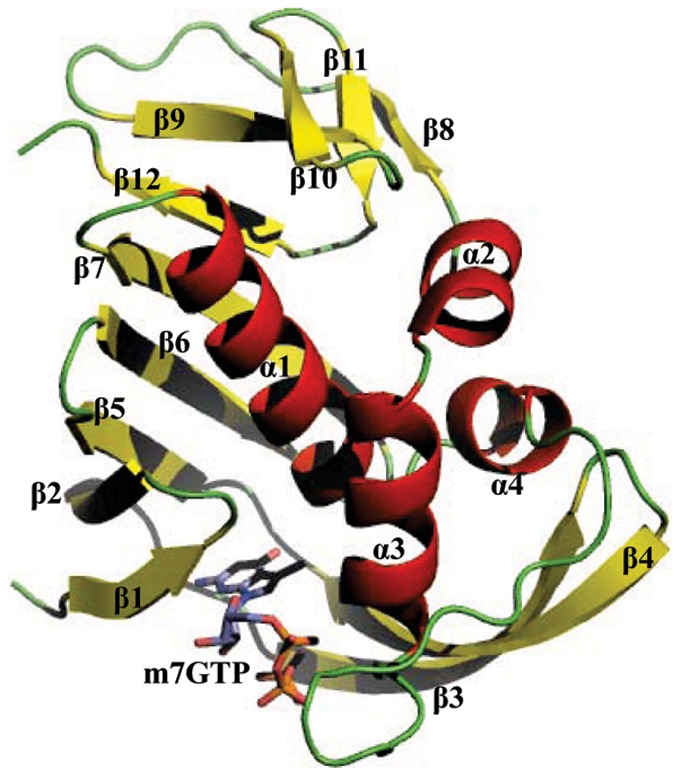
Illustratively showing the binding mode of the PB2 cap binding domain in the presence of the cap analog m7GTP. The protein structure was shown in a ribbon diagram, while m7GTP is in ball-and-stick representations. The secondary structure elements of the PB2 cap binding domain are labeled with α-helices in red and β-strands in yellow. The cap analog m7GTP is colored according to its atomic types.

The crystal structure of the influenza A virus PB2 subunit in the protein structure databases is the minimal cap binding domain (residues 318–483), in which a m7GTP molecule was also crystallized as a cap analog (Figure 1). During our MD simulations, RMS deviations for the Cα atoms of the PB2 cap-binding domain from the crystal structure were calculated (Figure S1), which was also believed as a crucial criterion for measuring the convergence of the simulation system concerned. For the current case, after about 800-ps MD simulations the PB2-m7GTP system reached to an equilibrated state with an average RMS deviation value of 1.55±0.18 Å from the crystal structure (starting structure), which at the end of the simulations was ∼1.53 Å. Additionally, the total energy of the PB2-m7GTP system in the equilibrated state was detected to be ∼6.742±0.017×10^4^ kJ/mol. All these information gives an indication that our MD simulations are quite credible and the PB2-m7GTP system has been equilibrated. Thus, the free energy calculations discussed below were then performed based on the equilibrated simulation trajectories.

**Figure 2:**
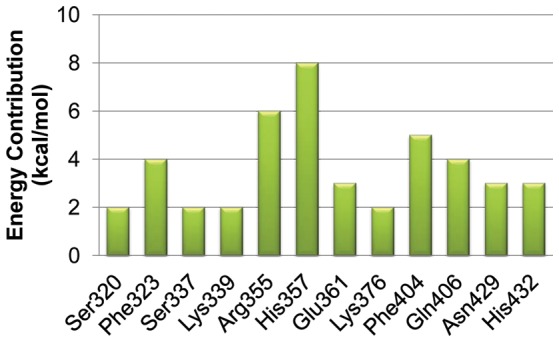
Energy contributions (kcal/mol) for each residue to the binding free energies of the PB2-m7GTP complex. Only the residues that have positive contributions to the binding free energies are listed in this figure. Among these residues, His357 has the most contributions owe to having both hydrogen bonding and π-π stacking interactions with the cap analog m7GTP.

**Figure 3:**
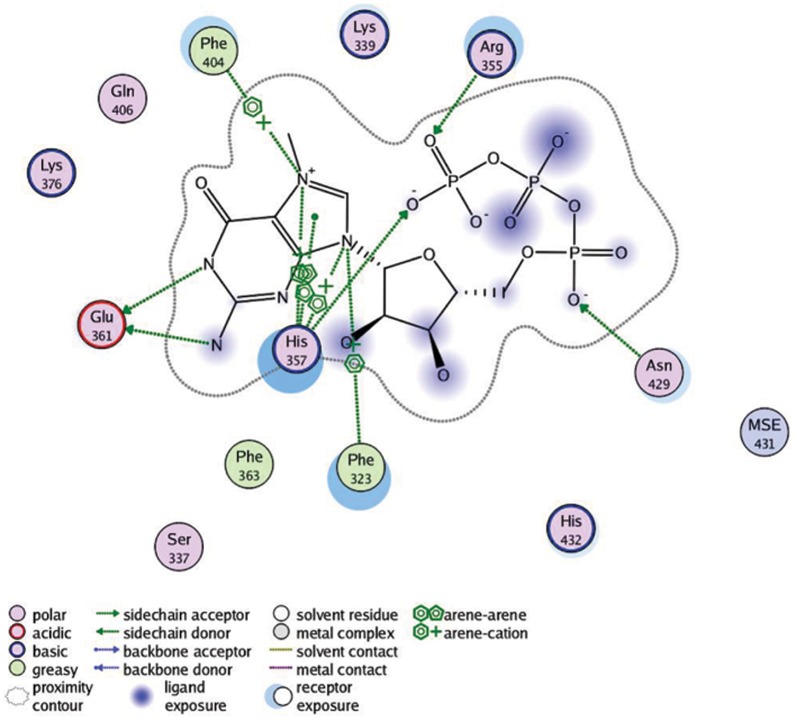
A 2D snapshot from MD simulations to show the binding interactions of the cap analog m7GTP with the key residues in the cap binding domain. The hydrogen bond is shown in green dashed line with the arrow pointed to the hydrogen acceptor. The π-π stacking and π-cation interactions are presented in green benzene ring symbol. The residues without any dashed line or benzene ring symbol have only van der Waals interactions with m7GTP.

In the current study, we selected both MM-PB/SA and MM-GB/SA approach, the most popular methods for the protein-ligand free energy calculations, to compute the binding free energies of the PB2-m7GTP complex. The binding free energyies for the PB2-m7GTP complex were −41.93±6.52 kcal/mol (MM-PB/SA) and −38.25±4.65 (MM-GB/SA), as shown in Table 1. The variance of the binding free energies obtained from MM-PB/SA and MM-GB/SA was mainly caused by the hydrophobic contributions to the solvation free energies and reaction field energies, which were calculated by either Possion Boltzmann or Generalized Born methods. Additionally, we also calculated the contributions to the binding free energies for each residue in the PB2 cap binding domain. As shown in Figure 2, the residues Ser320, Phe323, Ser337, Lys339, Arg355, His357, Glu361, Lys376, Phe404, Gln406, Asn429, and His432 had significantly positive contributions to the binding free energies of the PB2-m7GTP complex. Among these residues, His357 had the most contributions to the binding free energies of the PB2-m7GTP complex. This residue employed both hydrogen bonding and π-π stacking interactions with the guanine ring of m7GTP (Figure 3), which was regarded as the key factor for recognizing and positioning substrates of the PB2 cap binding domain.

**Table 1:** Binding free energies (kcal/mol) of m7GTP and other ligands calculated from both MM-PB/SA and MM-GB/SA.

Energy term	PB2-m7GTP	PB2-CPD1	PB2-CPD2
ΔE_ele_	−1048.00±27.60	−39.20±7.03	−25.42±4.99
ΔE_vdw_	−36.74±4.09	−42.17±3.41	−62.19±3.45
ΔE_gas_	−1084.75±27.16	−81.37±6.43	−87.62±5.17
ΔG_sur/PB_	−5.86±0.14	−5.68±0.16	−7.08±0.12
ΔG_cal/PB_	1048.68±25.03	51.05±4.98	55.58±3.83
ΔG_ele/PB_	0.67±7.86	11.85±5.43	30.16±4.41
ΔG_sol/PB_	1042.81±25.03	45.37±4.90	48.50±3.80
ΔG_bind/PB_	−41.93±6.52	−36.00±4.70	−39.12±4.20
ΔG_sur/GB_	−5.86±0.14	−5.68±0.16	−7.08±0.12
ΔG_GB_	1052.36±25.19	42.05±4.25	40.90±3.45
ΔG_ele/GB_	4.36±5.89	2.84±4.26	15.48±2.57
ΔG_sol/GB_	1046.50±25.19	36.36±4.21	33.82±3.44
ΔG_bind/GB_	−38.25±4.65	−45.01±3.95	−53.80±3.15

ΔE_ele_: non-bonded electrostatic energy+1,4-electrostatic energy.

ΔE_vdw_: non-bonded van der Waals energy+1,4-van der Waals energy 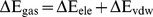

ΔG_sur/PB_: hydrophobic contributions to solvation free energy for the Possion Boltzmann calculations.

ΔG_cal/PB_: reaction field energy calculated by the Possion Boltzmann approach 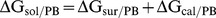 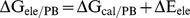 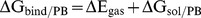

ΔG_sur/GB_: hydrophobic contributions to solvatoion free energy for the Generalized Born calculations.

ΔG_cal/PB_: reaction field energy calculated by the Generalized Born approach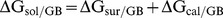 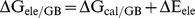 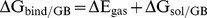

### Structural analysis of the PB2-m7GTP complex

According to the crystal study, the cap analog m7GTP was non-covalently bound in the cap binding domain of influenza A virus PB2 subunit. Thus, our analyses of the interactions of the PB2 cap binding domain with m7GTP were focused on the non-covalent binding interactions, such as hydrogen bonding interactions and hydrophobic interactions. In the current study, we continued to use the definitions in our previous studies to define hydrogen bonds and hydrophobic interactions [46], [47]. If the distance between the heavy atoms of a donor and an acceptor was less than 3.5 Å and the donor-H-acceptor angle was less than 30°, we thought that they could form a hydrogen bond. If the distance between the mass centers of the hydrophobic groups of the cap binding domain and m7GTP were less than 6.5 Å, we believed that they could have a hydrophobic interaction.

The detailed information of the hydrogen bonding interactions between the protein and m7GTP was described in Table 2. In Table 2, we only listed the information for the hydrogen bond having an occupancy more than 10%, which is believed to play crucial roles in the ligand binding. Hsi357, together with Ser320, Lys339, Arg355, Glu361, Lys376, and Gln406, formed an important hydrogen bonding interaction network, which was in agreement with the free energy calculations and believed to be essential for m7GTP binding and positioning. However, among the aforementioned residues, His357 was considered to be the key component by reason that it was detected to contribute two important hydrogen bonds. One was formed by the side-chain nitrogen (Nε_2_) of His357 and the oxygen (O6) on the β-phosphorus of m7GTP with an average distance of 3.95±0.60 Å, while another was by the backbone nitrogen (N) of His357 and ketonic the oxygen (O) from the guanine ring of m7GTP with an average distance of 2.97±0.30 Å. Notably, the hydrogen bond formed by His357 (N) and m7GTP (O6) had a comparatively highest occupancy (95.6%) among all the detected hydrogen bonds, functioning to position the guanine ring in a correct site. The hydrogen bonds formed by Ser320, Glu361, and Gln406 employed the similar function that they could keep the guanine ring of m7GTP in a correct direction and site in the active site. Otherwise, the hydrogen bonds formed by Lys339 and Arg355 were believed to play an important role in positioning the phosphorus tail.

**Table 2:** Detailed information for the important hydrogen bonding interactions between the PB2 cap binding domain and cap analog m7GTP.

Rank	Residues	m7GTP	Distance/Å	Occupancy
1	His357 (N)	O6	2.97±0.30	95.6%
2	Gln406 (N)	O1	3.01±0.16	87.2%
3	Ser320 (Oγ)	O5	3.10±0.59	72.0%
4	Arg355 (Nη_1_)	O4	3.18±1.08	64.0%
5	Lys376 (Nξ)	N2	3.36±1.02	60.0%
6	Glu361 (Oε_1_)	N3	3.51±0.63	31.6%
7	Lys339 (Nξ)	O7	3.61±0.65	22.7%
8	His357 (Nε_2_)	O6	3.95±0.60	12.9%

Besides the hydrogen bonding interactions, hydrophobic interactions also play key roles in substrate binding. In the current case, the guanine ring was the major hydrophobic group of the cap analog m7GTP, which could provide hydrophobic interactions with the key residues in the active site. Addition to forming two significant hydrogen bonds, His357 also had strong hydrophobic interactions with the guanine ring of m7GTP with a probability of more than 95%. Additionally, Glu361, Lys376, and Gln406 were also detected to have strong hydrophobic interactions in over 90% frames from the MD simulations, which could also form significant hydrogen bonds according to the aforementioned discussions. Arg355, a key residue for hydrogen bonding interactions of m7GTP, also employed a significant hydrophobic interaction with the guanine ring of m7GTP in nearly 50% frames along the simulation trajectories.

### Aromatic sandwich mechanism for the cap binding

How does the cap binding domain discriminate between capped RNAs and non-capped ones? This is of primary importance to the functions of the influenza A virus PB2 subunit. Besides the PB2 subunit, 3 cellular cap-binding proteins are well known by now: the eukaryotic translation initiation factor eIF4E [48], cap-binding complex (CBC [49]) consisting of 2 subunit cap-binding protein 20 (CBP20) in association with an ancillary protein CBP80, and the vaccinia viral protein 39 (VP39 [50]). Based on the discussions above and other cap binding proteins in the protein structure databases, we could get a direct impact on the active site of the PB2 cap binding domain, based on which 2 main features contributing to the cap-specific recognition were summarized.

**Figure 4:**
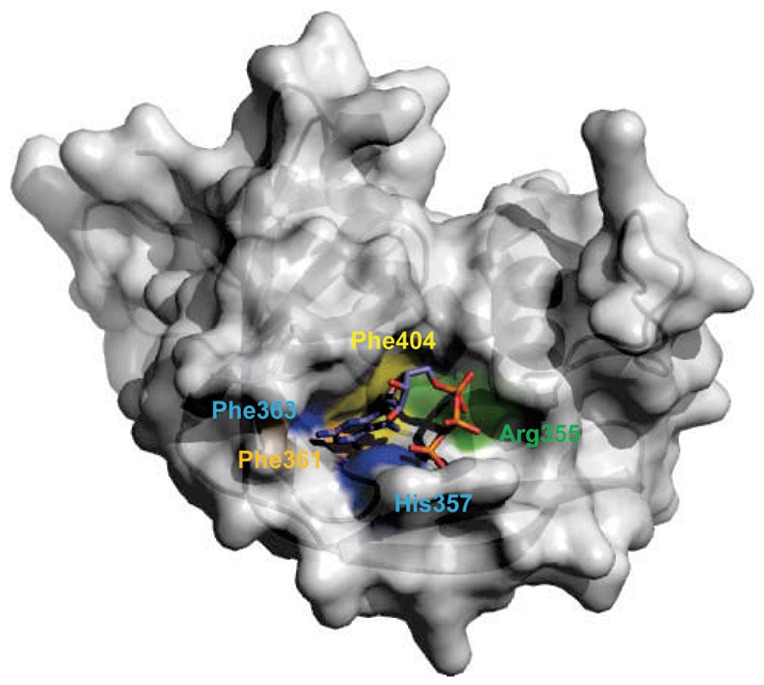
The cap-binding pockets for the influenza A virus PB2 subunit. The residues around the cap-binding pocket are colored so that those aromatic amino acids forming the cap sandwich around the cap analogue m7GTP are in blue, those binding the functional groups of the guanine residue are in orange, those stabilizing the 7-methyl group are in yellow and those binding the triphosphate moiety are green.

The binding pocket that accommodated the m7GTP aromatic ring was believed to stack between two aromatic residues Phe363 and His357, which were Trp56 and Trp102 in eIF4E, Tyr20 and Tyr43 in CBC, Tyr22 and Phe180 in VP39. Thus, the cap recognition and binding mechanism was also known as an aromatic sandwich mechanism, which had been detected from other small-molecule models by crystallographic and theoretical studies [51]–[54]. In the aromatic sandwich mechanism, the stacked aromatic rings employed strong π-electron interactions due to the perfect alignment of the aromatic rings of Phe363, His357 as well as m7GTP, and extensive overlap regions in the two stacked rings (Figure 4). This was why the cap-binding protein had >100-fold low affinity for non-methylated cap analogues [55]. Additional experiments showed that the mutation of the aromatic residues involved in the aromatic sandwich mechanism could reduce the binding affinities and specificities for cap analogues.

Residue Phe404 was of a crucial importance for delocalization of the positive charge from N7-methylation of m7GTP purine ring. The delocalization of the positive charge was also good for salt bridges and hydrogen bonds between N1 and N2 of m7GTP and the carboxylate groups of Glu361 in the active sites. The residues having similar functions with Phe404 were also found in eIF4E (Glu103), CBC (Asp116), and VP39 (Glu223). When these critical acidic residues were substituted by other amino acids, the affinity and specificity of the cap analogues significantly decreased [56]. Thus, Phe404 was involved in these additional contacts in the case of the influenza A virus PB2 subunit, although this residue was also involved in the aromatic stacking with m7GTP.

### Virtual screening for novel drugs

Based on the structural analysis of the binding mode for the PB2-m7GTP complex, we can obtain a deeper insight into the cap-binding pocket of the PB2 subunit. Based on this knowledge, we performed virtual screening on our in-house Finally, we found 5 candidates which might be potential leading compounds for the PB2 subunit, and their detailed information was listed in Table S1. Interestingly, Compound 1 (Cpd1) and Compound 2 (Cpd2) have been reported to have inhibitory activities against the influenza A virus PB2 subunit [57], [58]. In 2003, Hooker and his co-workers established a novel quantitative assay system to analysis the interactions between cap-binding proteins and cap analogues. Using this system, they have identified Cpd1 as a novel and selective inhibitor of the influenza cap binding protein [57]. Cpd2 is a phenethylphenylphthalimide analog derived from thalidomide, which is also proved to have strong inhibiting activity to the influenza virus cap-binding protein [58].

Their binding free energies calculated from MD trajectories by MM-PB/SA and MM-GB/SA were listed in Table 1, from which we found that Cpd1 and Cpd2 had a comparatively higher binding affinity (−45.01±3.95 kcal/mol and −53.80±3.15 kcal/mol respectively obtained from MM-GB/SA) than that of m7GTP (−38.25±4.65 kcal/mol). The binding affinity calculated from MM-PB/SA for Cpd2 (−7.08±0.12 kcal/mol) was also higher than that of m7GTP (−5.86±0.14 kcal/mol). However, the one obtained from MM-PB/SA for Cpd1 (−5.68±0.16 kcal/mol) was a little lower then that of m7GTP. This was mainly caused by the free energy calculation method. Due to having a number of negative charges in the phosphorus tail, the electrostatic contribution (ΔG_ele/PB_) to solvation free energy for m7GTP might be not correctly computed. In the current case, the electrostatic contribution to solvation free energy for m7GTP was 0.67±7.86 kcal/mol using MM-PB/SA, while those for Cpd1 and Cpd2 were 11.85±5.43 kcal/mol and 30.16±4.41 kcal/mol, respectively.

Additionally, Cpd1 and Cpd2 had significantly higher van der Waals potential energies (−42.17±3.41 kcal/mol and −62.19±3.45 kcal/mol respectively) that that of m7GTP (−36.74±4.09 kcal/mol). Detailed analyses showed that His357, Phe404 and Gln406 contributed strong hydrophobic interactions to Cpd1 and Cpd2 in over 90% frames along the simulation trajectories. According to the previous analysis, these residues also had significant hydrophobic interactions with m7GTP. In the PB2-Cpd1 complex, Met431 had also a strong hydrophobic interaction in more than 90% frames, which in the PB2-m7GTP and PB2-Cpd2 complexes were in 40% and 60% frames. Additions to the hydrophobic interactions, some residues, such as Arg355, Glu361 as well as Gln406 in the PB2-Cpd1 complex and Arg379 and Glu407 in the PB2-Cpd2 complex were also detected complexes to have important hydrogen bonding interactions.

### Multi-target selectivity study

To further study the multi-target selectivity of the 5 candidates mentioned above, we also docked the candidates into the 841 known targets from the Potential Drug Target Database (PDTD) [45]. Based on their therapeutic areas, these targets can be categorized into 14 different types: Heoplastic disease, hormones and hormone antagonists, viral infections, blood and blood-forming organs, immunomodulation, bacterial infections, renal and cardiovascular functions, synaptic and neuroeffector junctional sites and central nervous system, parasitic infectious disease, inflammation, the vitamins, gastrointestinal functions, fungal infections and uterine motility. The docking results were ranked according to their docking scores, and the top 5 candidate-binding proteins were listed in Table S2. Interestingly, the top hit of the multi-target selectivity for all the 5 candidates were the influenza virus cap-binding domain PB2 subunit (2vqz.pdb), indicating that influenza virus cap-binding domain PB2 subunit might be the preferred target for these candidates with respect to the other targets approved by the US FDA. Additionally, the top 5 binding proteins for all the candidates were involved in the viral infections according to the PDTD category.

In summary, influenza A virus PB2 subunit is responsible for replication and transcription of the viral RNA in the nucleus of infected cells. The initial step of viral mRNA transcription is the host-cell pre-mRNA bound to the PB2 subunit by its 5′ caps. How the cap-binding domain discriminates between capped RNAs and non-capped ones is of primary importance to the functions of the influenza A virus PB2 subunit. In the current study, we performed molecular dynamics simulations and free energy calculations on the influenza A virus PB2 subunit with a cap analog m7GTP. Based on the simulation trajectories and structural analyses, we identified some key residues, such as Arg355, His357, Glu361, and Gln406, to have significant hydrogen bonding and hydrophobic interactions with the guanine ring of m7GTP, recognizing and positioning the guanine ring with an aromatic sandwich mechanism, which would be the key strategy for searching and designing novel inhibitors against the influenza A virus PB2 subunit. Thus, we applied this idea to search our in-house database, and found 5 candidate structures that might employ significant inhibitory activities to the PB2 subunit. Interestingly, two of the candidates (Cpd1 and Cpd2) obtained from the virtual screening procedure had been reported to have inhibitory activities against the influenza virus cap-binding proteins. The free energy calculations for both PB2-Cpd1 and PB2-Cpd2 showed that they employed comparatively higher binding affinities with the PB2 subunit than that of m7GTP due to having much stronger non-bonded van der Waals energies. We believed that our findings could provide a deeper understanding of the cap recognition for the influenza A virus, offering useful information for searching or designing novel drugs against influenza A virus.

## Supporting Information

Figure S1 **The time-dependent RMS deviations for the Cα atoms of the PB2 cap-binding domain from the crystal structure.** PB2-m7GTP system reached to its equilibrated state after about 800-ps MD simulations with an average RMS deviation value of 1.55±0.18 Å from the crystal structure, which at the end of the simulations was ∼1.53 Å.
(TIF)

Table S1 **The candidates obtained from virtual screening using m7GTP as a structural template and aromatic sandwich mechanism as a designed strategy.**
(DOC)

Table S2 **The top 5 binding proteins of each candidates derived by the multi-target selectivity study.**
(DOC)
