# Supplementary material for: Computational Studies on the Substrate Interactions of Influenza A Virus PB2 Subunit
Source: PLoS One. 2012 Sep 5;7(9):e44079. doi: 10.1371/journal.pone.0044079 (PMC3434214; doi:10.1371/journal.pone.0044079)
Supplement: Table S1 — The candidates obtained from virtual screening using m7GTP as a structural template and aromatic sandwich mechanism as a designed strategy. (DOC) [file pone.0044079.s002.doc]

**Table S1.** The candidates obtained from virtual screening using m7GTP as a structural template and aromatic sandwich mechanism as a designed strategy.

| Rank | Structure | Formula | NSC No. | CAS No. |
| --- | --- | --- | --- | --- |
| 1 |  | C_16_H_20_N_5_O_2_ | — | — |
| 2 |  | C_22_H_13_Cl_4_NO_4_ | — | — |
| 3 |  | C_16_H_16_N_4_O_3_ | 140050 | 76628-78-9 |
| 4 |  | C_22_H_10_Cl_2_N_2_O_4_ | 58280 | 6626-72-8 |
| 5 |  | C_25_H_17_O_6_ | 245423 | 500884-25-3 |
