# Supplementary material for: Computational Studies on the Substrate Interactions of Influenza A Virus PB2 Subunit
Source: PLoS One. 2012 Sep 5;7(9):e44079. doi: 10.1371/journal.pone.0044079 (PMC3434214; doi:10.1371/journal.pone.0044079)
Supplement: Table S2 — The top 5 binding proteins of each candidates derived by the multi-target selectivity study. (DOC) [file pone.0044079.s003.doc]

**Table S2.** The top 5 binding proteins of each candidates derived by the multi-target selectivity study.

| Candidates | Rank | PDB ID | Docking Score | Target Name |
| --- | --- | --- | --- | --- |
|  | 1 | 2vqz | -8.03 | Influenza virus cap-binding domain PB2 subunit |
|  | 2 | 2sim | -5.23 | Neuraminidase |
|  | 3 | 7hvp | -1.08 | HIV Protease |
|  | 4 | 1d6a | 1.98 | Pokeweed Antiviral protein |
|  | 5 | 1e2k | 2.36 | Thymidine Kinase |
|  | 1 | 2vqz | -11.32 | Influenza virus cap-binding domain PB2 subunit |
|  | 2 | 1dxp | -7.56 | Nonstructural protein NS2 |
|  | 3 | 1iam | -6.14 | Intercellular Adhesion Molecule-1 |
|  | 4 | 1ida | -4.87 | HIV-2 Protease |
|  | 5 | 1kim | -1.63 | Thymidine Kinase |
|  | 1 | 2vqz | -10.36 | Influenza virus cap-binding domain PB2 subunit |
|  | 2 | 1in4 | -8.76 | Holiday Junction DNA Helicase Ruvb |
|  | 3 | 1hvl | -6.25 | HIV Protease |
|  | 4 | 1jma | -5.46 | Herpes simplex virus glycoprotein D |
|  | 5 | 1uk4 | -3.39 | SARS Coronavirus Main Proteinase |
|  | 1 | 2vqz | -10.51 | Influenza virus cap-binding domain PB2 subunit |
|  | 2 | 1tdb | -6.98 | Thymidylate Synthetase |
|  | 3 | 1zfj | -5.24 | Inosine-5’-monophosphate dehydrogenase |
|  | 4 | 1uk4 | -3.86 | SARS Coronavirus Main Proteinase |
|  | 5 | 1kim | -1.25 | Thymidine Kinase |
|  | 1 | 2vqz | -12.07 | Influenza virus cap-binding domain PB2 subunit |
|  | 2 | 1in4 | -8.98 | Holiday Junction DNA Helicase Ruvb |
|  | 3 | 4hmg | -7.21 | Hemagglutinin |
|  | 4 | 1zfj | -5.56 | Inosine-5’-monophosphate dehydrogenase |
|  | 5 | 1jma | -4.03 | Herpes simplex virus glycoprotein D |
